# Can mHealth improve timeliness and quality of health data collected and used by health extension workers in rural Southern Ethiopia?

**DOI:** 10.1093/pubmed/fdy200

**Published:** 2018-12-14

**Authors:** W Mengesha, R Steege, A Z Kea, S Theobald, D G Datiko

**Affiliations:** 1 REACH Ethiopia, Hawassa, Ethiopia; 2 Department of International Public Health, Liverpool School of Tropical Medicine, Pembroke Place, Liverpool L3 5QA, UK

## Abstract

**Background:**

Health extension workers (HEWs) are the key cadre within the Ethiopian Health Extension Programme extending health care to rural communities. National policy guidance supports the use of mHealth to improve data quality and use. We report on a mobile Health Management Information system (HMIS) with HEWs and assess its impact on data use, community health service provision and HEWs’ experiences.

**Methodology:**

We used a mixed methods approach, including an iterative process of intervention development for 2 out of 16 essential packages of health services, quantitative analysis of new registrations, and qualitative research with HEWs and their supervisors.

**Results:**

The iterative approach supported ownership of the intervention by health staff, and 8833 clients were registered onto the mobile HMIS by 62 trained HEWs. HEWs were positive about using mHealth and its impact on data quality, health service delivery, patient follow-up and skill acquisition. Challenges included tensions over who received a phone; worries about phone loss; poor connectivity and power failures in rural areas; and workload.

**Discussion:**

Mobile HMIS developed through collaborative and locally embedded processes can support quality data collection, flow and better patient follow-up. Scale-up across other community health service packages and zones is encouraged together with appropriate training, support and distribution of phones to address health needs and avoid exacerbating existing inequalities.

**Keywords:**

CHWs, equity, ethics, Ethiopia, Health Management Information system, HEP, maternal health, mHealth, TB

## Resumen

**Antecedentes:**

Los agentes de extensión sanitaria (AES) son el equipo clave dentro del Programa de Extensión Sanitaria de Etiopía, llevando la atención para la salud a las comunidades rurales. Las directrices de las políticas nacionales apoyan el uso de mSalud para mejorar la calidad y el uso de la información. Informamos sobre un sistema de Información para la Administración de la Salud móvil con los AES y evaluamos su impacto en el uso de la información, la provisión de servicios de salud comunitaria y las experiencias de los AES.

**Metodología:**

Usamos un enfoque de métodos mixtos, incluyendo un proceso de desarrollo de la intervención reiterado para dos de los 16 paquetes de servicios de salud esenciales, análisis cuantitativo de las nuevas inscripciones e investigación cualitativa con los AES y sus supervisores.

**Resultados:**

El enfoque reiterado respaldó la responsabilidad del personal de la salud con respecto a la intervención y se registraron 8.833 clientes en el sistema móvil de Información para la Administración de la Salud por medio de 62 AES capacitados. Los AES mostraron una actitud positiva en cuanto al uso de mSalud y su impacto en la calidad de los datos, la prestación de los servicios de salud, el seguimiento con el paciente y la adquisición de capacidades. Los desafíos incluyeron conflictos con respecto a quién recibió un teléfono, preocupación por perder dicho aparato, los apagones, conectividad y el volumen de trabajo.

**Debate:**

El sistema móvil de Información para la Administración de la Salud integrado, desarrollado mediante procesos de colaboración e inserción en el marco local, puede respaldar la recolección y el flujo de datos fidedignos y un mejor seguimiento con el paciente. Se alienta la ampliación transversal con otros paquetes de servicios para la salud comunitaria y zonas, junto con la capacitación adecuada, el respaldo y la distribución de teléfonos para abordar las necesidades de salud y evitar incrementar las desigualdades existentes.

**Palabras clave:**

mSalud, género, agentes de extensión sanitaria, ética, Etiopía, sistema de Información para la Administración de la Salud, salud materna, tubérculo, y comunidades.

## Résumé

**Contexte:**

Les agents de vulgarisation sanitaire (AVS) sont le cadre clé du programme éthiopien de vulgarisation sanitaire qui étend les soins de santé aux collectivités rurales. L’orientation stratégique nationale appuie l’utilisation de santé mobile pour améliorer la qualité et l’utilisation des données. Nous présentons des évidences sur un système mobile de gestion de l’information sur la santé avec les AVS et évaluons son incidence sur l’utilisation des données, la prestation de services de santé communautaires et l’expérience des AVS.

**Méthodologie:**

Nous avons utilisé une approche à méthodes mixtes, y compris un processus itératif d’élaboration d’interventions pour 2 des 16 forfaits de services de santé essentiels, une analyse quantitative des nouvelles homologations, et une recherche qualitative avec les AVS et leurs superviseurs.

**Résultats:**

L’approche itérative appuyait la prise en charge de l’intervention par le personnel de la santé, et 8 833 clients étaient inscrits au système mobile de gestion de l’information sur la santé par 62 AVS formés. Le recours à la santé mobile ainsi que son incidence sur la qualité des données, la prestation des services de santé, le suivi des patients et l’acquisition de compétences était favorisé par les AVS. Les difficultés comprenaient des tensions par rapport à qui recevait le téléphone, des inquiétudes au sujet de la perte du téléphone, les pannes de courant, la faible connectivité et la charge de travail.

**Discussion:**

Un système mobile de gestion de l’information sur la santé mis au point grâce à des processus collaboratifs et intégrés localement peut appuyer la collecte et l’acheminement de données de qualité ainsi qu’un meilleur suivi des patients. On encourage l’expansion à d’autres forfaits et zones de services de santé communautaires, ainsi que la formation, le soutien et la distribution appropriés de téléphones pour répondre aux besoins de santé et éviter d’exacerber les inégalités existantes.

**Mots clés:**

santé mobile, agents de vulgarisation sanitaire, étique, Éthiopie, système de gestion de l’information sur la santé, santé maternelle, tuberculose, genre et collectivités.

## Introduction

To ensure universal access to primary health care at community level, Ethiopia launched its flagship health programme known as the Health Extension Programme (HEP) in 2003.^1,2^ The programme was designed to provide equitable access to primary health care by bringing services to the community. Female community health workers, referred to as health extension workers (HEWs), are the key cadre delivering the HEP packages of services.^1,3^ They are recruited from the local community, which is shown to improve relationships within communities,^3–5^ have completed school until at least grade 10, and received training for 1 year to provide community-based services.^6^ HEWs work at health posts based in ‘kebeles’ (the smallest administrative unit). Two HEWs are assigned in each kebele with an average population of 5000 people (~1000 households). They devote 75% of their time to making house-to-house visits.^3^

Tuberculosis (TB) and poor maternal health (MH) outcomes contribute to high levels of morbidity and mortality in Ethiopia^2^ and therefore are national public health priority areas included as two of the 16 packages delivered by HEWs. Since the implementation of HEP, coverage of health services has improved. Maternal delivery coverage rates reached 72.7% and the TB detection rate reached 61.3% in 2016.^7^ Improving MH outcomes and effective TB care requires early identification and follow up, linkage to community or facility-based services, and improved data use, which can be acted upon to support more equitable service delivery.

HEWs use the Health Management Information system (HMIS) to collect data at the health post, which are routinely fed up to the health centre for validation, compilation and further reporting.^6^ These data are used to track health outcomes and plan the use and allocation of resources at community level. The HMIS relies on paper-based reports, transported upwards from health posts to health centres and higher levels. Key challenges to the HMIS include delays in reporting, incompleteness or inconsistency of data, inadequate data collection tools and poor monitoring systems. This results in poor data use, delays in patient follow-up, inability to take timely action and missed opportunities for responsive planning.

In 2012, the Ethiopian Ministry of Health developed a mobile Health (mHealth) (eHealth refers to the use of ICT, especially (but not only) the Internet to enable health and health care.^8^ mHealth is the use of mobile phones and any other communication devices for health services.) strategy providing a framework for action.^6,9^ The framework discusses how HEWs, being the first port of call for remote and rural populations, should be the drivers for the first mHealth roll out phase.^9^ The expectation is that improving data collection and reporting by HEWs will improve the quality and timeliness of data for local decision-making, and consequently increase accountability, transparency and redress inequities in the health system.^9^

In response to the mHealth strategy and to the central role of HEWs in providing more equitable and responsive health services, we designed and implemented an integrated mHealth intervention (The intervention was part of a programme funded by the International Development Research Centre (IDRC) and called Strengthening Equity through Applied Research Capacity Building in eHealth (SEARCH). The SEARCH project ran from 2014 to 2017 and built on prior work that began in 2010 under the TB REACH project implemented by REACH Ethiopia.) using mobile phones to support timely capture of quality data by HEWs to improve the effectiveness and equity of primary health care service provision.^2,10^ The aim of the present study was to assess the impact of the intervention on quality of data collection and reporting for TB and MH, as well as to examine experiences of HEWs using the technology.

### Methodology

The study was a mixed method evaluation. Data were collected via three complementary processes: learning from the iterative process of intervention development and problem solving; quantitative data tracking on the coverage and uptake of the mobile HMIS; and qualitative data collection to examine the impact the technology had on HEWs’ experiences.

### The intervention

The project was conducted in Sidama zone, Southern Ethiopia, a densely populated zone with ~3.5 million people^6,10^ and ran from July 2013 to July 2017. The population level access to mobile phones is improving and most health workers and policy makers own their own phones. There is adequate mobile connectivity in the area (though it can suffer connectivity failures) and most health facilities have a power supply. The project was implemented in six Primary Health Care Units (PHCU) based in six districts, with a minimum of four and a maximum of seven health posts each. They were selected based on their patient load, and via discussion with health system stakeholders. We identified health facilities that had problems with the HMIS and patient follow-up.

Prior to the intervention, HEWs relied on a paper-based HMIS. Informed by a baseline analysis,^6^ we designed and established a mobile HMIS for HEWs to register clients for TB and MH services using mobile smartphones. The intervention involved providing smartphones to health workers (HEWs, heads of health centres and focal persons from the district and zonal levels), with a preloaded data entry platform, via an adapted CommCare platform^11^ (see Appendix 4). The mobile HMIS intervention was implemented alongside the existing paper-based HMIS. Programme managers, health workers and HEWs were trained on TB and MH, data collection and entry onto the platform; how to use the reminder text messages and client follow-up.

The mobile HMIS was developed in close collaboration with the Ministry of Health and using the Ethiopian mHealth strategy as a guiding framework. The use of open standards and open source software system enabled more seamless and secure integration within existing HMIS formats and data flow. A total of 97 smartphones were distributed to HEWs, and eight computers to health centres in the implementation districts. Sensitization and training was conducted with 126 stakeholders at different levels of the health system—including focal persons for TB, MH and HMIS, HEW supervisors, heads of health centres, districts and all 62 HEWs from the PHCUs. Data entered was anonymized and uploaded to a central database accessible at different levels.

The mobile HMIS enables HEWs to register clients directly onto the mHealth platform. For each client, data are captured about sociodemographic details, symptoms and duration, laboratory results, treatment, follow up and outcomes (for TB clients) and information on gravidity, parity, gestational age, laboratory examination for ANC, delivery and its outcome and post-natal care (for MH clients). To ensure completeness of data, inbuilt data validation was included so that fields cannot be left blank.

**Fig. 1:**
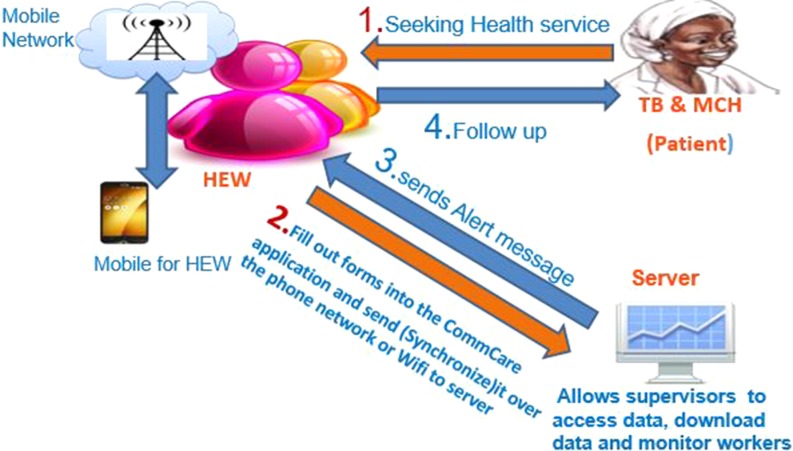
Flow diagram of the intervention process.

Data was stored and uploaded to the cloud when network connection was available and immediately synchronized to the HMIS. This helped supervisors and heads of health centres track and analyse data. Reminder messages were sent to HEWs and programme focal persons with the aim of reducing the number of patients who may be lost to follow up (Fig. 1). The messages included reminders for ANC or TB laboratory tests and due dates for delivery. A minimum of three messages were sent per client.

## Methods

### Iterative learning

The intervention had a strong focus on training and sensitization to lay the foundation for sustained positive change among female HEWs. An engagement strategy was used to ensure ongoing communication and problem solving, for example, through the setting-up of a technical working group and district and catchment level meetings. Regular activities enabled dialogue and feedback on ongoing challenges and opportunities emerging from the project, including:

- Training and sensitization workshops with health workers and key stakeholders to create awareness of the project aim and related processes.
- Regular supportive supervision of HEWs alongside a review of their activities.
- Ongoing stakeholder meetings held at all levels of the intervention in the community.
- Regular HEW meetings to discuss the project, share experiences and highlight ways to improve project performance.

Structured notes were taken from these different interactions, which were used to frame and support our analysis (see Appendix 1).

### Quantitative tracking of impact

The maternal health and TB registration data captured via smartphones were immediately available for analysis once uploaded to the main server and was tracked from December 2015 to July 2017. The anonymized data were routinely downloaded by the IT professional of the project (W.M.), exported to an excel sheet, and then checked and analysed to monitor numbers of new registrations and alert messages sent. These data were cross checked with the existing paper-based HMIS for regular updates, completeness, the number of follow-up messages received, and action was taken to ensure follow up if needed. Server-based and paper-based data were used to evaluate mHealth activity. When network problems arose, the paper register was used to fill gaps.

### Understanding HEW experiences

Qualitative methods included face-to-face semi-structured interviews (*n* = 19) and focus group discussions (FGDs) (*n* = 8) with a mix of HEWs, supervisors and community leaders (Table 1). Interviews were conducted in four of the intervention districts purposively selected for variation in geographic location and performance.

**Table 1:** Qualitative interviews conducted by participant, district and sex

District participant	District 1	District 2	District 3	District 4
HEW	2 × IDIs (Female) 1 × FGD (Female)	3 × IDIs (Female) 1 × FGD (Female)^a^	4 × IDIs (Female)	5 × IDIs (Female) 1 × FGD (Female)
HEW supervisor	1 × IDI (Female) 1 × IDI (Male)	1 × IDI (Male) 1 × FGD (Male)^b^	1 × IDI (Male) 1 × FGD (Male)	1 × IDI (Male) 1 × FGD (Male)
Community leaders	1 × FGD (Male)	1 × FGD (Male)	1 × FGD (Male)	1 × FGD (Male)

FGD = focus group discussion; IDI = in depth interview.

^a^Merged with participants from District 3.

^b^Merged with participants from District 1.

Interview topic guides addressed if and how smartphones helped or hindered the HEWs’ role; use of the phones outside of work; and changes in relationships between and among health staff since the introduction of the smartphones. A female research assistant, fluent in Sidamigna (local dialect) was recruited and trained over two days. The lead qualitative researcher (R.S.) was present during the interviews to clarify any questions or concerns. Interviews were conducted at health posts and health centres within private areas, at a time convenient to the respondents. Following informed consent, interviews were recorded using digital Dictaphones.

The recordings were transcribed verbatim and translated to English by experienced researchers. The quality of translation was checked by a member of REACH Ethiopia (A.Z.K) using sample transcripts. Transcripts were read and re-read by the lead researcher (R.S.), informing the development of codes for analysis, identifying emerging themes and areas for further exploration through the iterative development of a coding framework in NVivo.^12,13^

#### Ethics statement

Ethical approval was given from the Federal Ministry of Science and Technology, National Research Ethics Review Committee in April 2014. A support letter was obtained from Regional Health Bureau to conduct the interviews. Written informed consent was obtained from the participants of the study.

## Results

### Increased accountability

Accountability occurs at different levels within the health system; from the HEWs to the communities they serve; from the supervisors to the HEWs; and from the supervisors and colleagues at the health centre to the district and regional level. The iterative approach taken ensured buy-in from all stakeholders and a sense of responsibility to account for one’s actions. This led to interest and accountability in the mobile HMIS, evidenced by continued discussion and engagement at various meetings throughout the project (see Appendix 1). Data can now be collected in real-time and is accessible throughout all levels of the health system through different reports and dashboards (see Appendices 2 and 3 for more information on reporting and information flow). Interviews with stakeholders suggest that this aided decision making and has helped create stronger links with policy makers for action.

It helps to hold clients’ information, to disseminate the necessary information from top to bottom and vice versa, it also helps for decision making purposes by relying on the data, to track the progress of the service and it also helps as a reminder to give service... [Supervisor, IDI]

Supervisors, who also receive the alert messages, indicated that the alerts encourage their active participation in patient follow-up and community leaders expressed a wish to receive the phones to aid follow-up in communities.

There is good motivation toward [the HEWs’] work. Additionally, when they receive the message alarm for a particular woman after entering data in mobile, they feel great satisfaction. This is common not only for health extension workers but also us. [Supervisor, FGD]

It is advisable to provide the mobile phone for all of us to give more services for pregnant women as well as for the community. [Community leader, FGD]

HEWs reported the mHealth system improved in the speed of preparing and delivering messages to their clients. The use of smartphones for prompting appointments was triangulated with similar responses from health professionals. Health-centre heads and kebele administrators indicated that use of smartphone reminders, if extended to kebele administrators, may reduce the number of non-attendees at clinical appointments. The main challenges reported by all respondents was the lack of internet connectivity and power failures in rural areas. These issues were solved by supervisors travelling to nearby areas with the mobiles where connectivity and power could be reached.

### Registrations and population reached

Prior to the project intervention, only paper-based recording and reporting existed; while delays and inconsistencies in reporting were common. Compared to the baseline, data on TB and maternal health have improved in accuracy, completeness and timeliness, as confirmed by multiple respondents.

There is an improvement in data quality as compared to previous years. ……[P]reviously when pregnant woman come to first/second visit of antenatal care, we register her information and wait several days without reporting [her]. But now we report daily performance within 2–3 h to multiple sites…This also helps to avoid data fallacy/wrong reports…and avoid missing to report. [HEW, FGD]

The data quality is improving for instance, when they feed data and miss something it is difficult to continue to the next steps unlike in hard copies. [Supervisor, FGD]

In past years, we were giving report to woreda [district] without performing the actual work in that particular kebele and this was cheating the community. But now this mobile helps to keep and report actual work that is done. [Community leader, FGD]

The 62 HEWs involved in the project worked with over 200 000 rural women and men. This population benefited from more targeted health services resulting from higher quality and more timely use of data collected. Increases were observed in the number of pregnant women identified, presumptive cases referred, and TB cases detected over time, resulting in improved follow-up and increased service uptake. The number of clients registered for skilled delivery increased every reporting period (Table 3), demonstrating increased enrolment in the system.

There are differences over time in numbers of ANC visits scheduled through the mHealth system. These discrepancies in reporting can be attributed to the fact that some pregnant women may not reach ANC4, possibly due to delayed initiation of ANC and delivery before reaching the fourth visit. In addition, some woman who do not follow ANC still deliver at health facilities. Qualitative research with HEWs and supervisors also revealed variations in reporting due to the nomadic nature of people in some districts, improved family planning services and reduced falsification of data. Collectively these can explain improvements in the accuracy of this measure and decreases in registrations for ANC and delivery as shown from baseline (Table 2) to endline (Table 3).

Yes, there is a reduction in the performance especially institutional delivery…The reason for this is most people in our kebele…frequently move around…and, there is a tendency to inflate reports. There is a change in attitude following the start of using this mobile. The data being fact makes the number reported less. [Supervior, FGD]

We faced a problem after we began using this technology. The health centre head and kebele focal asked that reason for the decline of our kebele antenatal care performance. If the coverage of family planning is high, it is obvious that the antenatal coverage is low. We informed them the reason behind for the decline of the service is due to the improvement of data quality. [HEW, IDI]

**Table 2:** Sidama zone (intervention zone) report at baseline (July 2013–June 2014)

Module	July ‘13–Sept ‘13	Oct ‘13–Dec ‘13	Jan ‘14–March ‘14	April ‘14–June ‘14	Total
ANC	1092	1083	1050	1060	4285
TB cases	66	78	72	38	254
Delivery	465	554	699	723	2441

**Table 3:** Sidama zone (intervention zone) cumulative report during the project period (December 2015–July 2017)

Module	Dec ‘15–April ‘16	Dec ‘15–Sept ‘16	Dec ‘15–Feb ‘17	Dec ‘15–July ‘17
ANC	1009	2072	2132	2409
TB cases	190	173	295	333
TB symptomatic cases	157	455	486	510
Delivery	981	1909	2590	2635
Post-natal care	901	1098	2076	2410

#### Alert messages

At baseline, health officials did not receive alert messages. Registration onto the mHealth platform triggers alert messages so HEWs can follow up with clients and reduce those who are lost to follow-up. About 2 700 alerts had been sent for MCH and 500 for TB by the end of July 2017 (Table 4).

**Table 4:** Total number of text alerts received by the HEW, for maternal health and TB services by district at project endline

	No. of ANC alerts sent	No. of Skilled delivery alerts sent	No. of TB alerts sent
Total	1236	1464	500

Follow-up alerts may directly benefit patients who may have otherwise been lost to follow-up due to health system challenges (no follow up/poor record keeping) or inequities that prevent access to care (by disability, geography, gender inequities and/or financial constraints). Qualitative interviews revealed that the follow up alerts that serve as reminders to the HEWs (and their supervisors) enable them to follow up with clients in a timely fashion, promoting equitable and responsive continuity of care.

The mobile phone is helping us to give pregnant women the necessary continuity of care such as antenatal care…It decreases…dropout rate. [Supervisor, FGD]

After training and handling this mobile I followed many mothers through mobile message and this makes me so happy. Because previously there were occasions where I forget to follow up of mothers for several reasons. [HEW, FGD]

They are identifying pregnant women and are feeding the data easily, it eases their work burden and we are following pregnant women to get continuity of care. [Community leader, FGD]

Conversely, this method of active follow-up can lead to ethical dilemmas. For example, clients may have reasons for not wanting to follow an approved course of action (e.g. giving birth in a delivery centre), which should be understood, discussed and respected. HEWs need to be appropriately supported in these situations.

Reflections from the iterative learning process revealed that during the reporting period, HEWs’ capacity to use the software improved. Taking a step back, it is important to explore if and how supplying mHealth technology to the all-female HEW cadre poses ethical issues, including putting them at risk of assault, theft or placing additional professional burdens (e.g. workload).

### HEWs’ experiences

#### Smartphones as a support

From qualitative interviews the main benefits for the HEWs pertain to skill building and increased respect from community members. Overall, HEWs viewed the technology as an aid to their work; enhancing accuracy of reporting, motivation, skill-building and confidence and support via the SMS reminders.

I became motivated. For example, previously if data of particular mother registered and reported once in a month, but now it helps us to follow up the mothers as TB cases [so] they do not miss their appointment date. [HEW, IDI]

[The phone] is good to work. For example, I may miss the appointment date of pregnant woman when I have work burden…but the mobile reminds me by the alarm… [HEW, IDI]

The intervention has also provided an opportunity to upgrade HEW skills and knowledge. Those with a smartphone at the health post reported increased participation in district level meetings, compared to control districts. These consequences may indirectly benefit individual HEWs’ career advancement opportunities as further training is offered to best performers, however, we were unable to measure this impact.

For example, I have gained knowledge on the mobile and it makes me able to do quality work and this increased my participation in different meetings. [HEW, IDI]

HEWs—regardless of phone ownership—reported receiving recognition from the community. However, one HEW reported that with this comes more expectations from the community. This requires exploration; the technology may add additional pressure, such as stress from feeling unable to meet expectations, and increased workload for HEWs.

#### Ethical concerns for HEWs

The phones were only given to one HEW per health post to avoid duplication of data, however, only one participant reported understanding this to be the reason for this allocation. HEWs who did not receive phones felt discouraged that they were not upgrading their skills in the same way: despite taking part in the initial training, they did not have opportunity to practice using the phones.

The organization provided the phone for routine work but she considers it as her own property since it was given free of charge. I am identifying pregnant women using the phone, but I am not practicing on it. [HEW, IDI]

Supervisors also suggested this could lead the HEWs without the phone evading mHealth related reporting, which may inadvertently add to the workload of the HEW with the smartphone, and cause tensions.

Since the other HEW is observing her colleague using [the phone] for purposes other than routine work she may not have good feeling. The one who doesn’t have the phone thinks that other HEW may get incentives…[and] may consider herself as inferior to her colleague and she might also say the mobile health issues are not my concern. [Supervisor, FGD]

Notwithstanding this, most participants did not report problems working together and some reported relying on their supervisors to help fill in any skill gaps with regards to using the technology.

One area of consideration that will need to be addressed if the technology is rolled out across all essential health packages is the duplication of tasks. Participants reported that they must spend time inputting the data into the phones in addition to fulfilling paper-based reporting duties. Others however expressed that this additional work burden was acceptable given the benefits to community members and themselves.

R2:… we are supposed to register information in mobile and this caused too much work burden as it takes time to feed information both in mobile and register. I consider it as simply a fashion but not to help community…I feel it added only work burden as we [only] register 10–15 mothers in daily basis.

R3: Even though it causes work burden; it is important as we are helping the mothers through it. [HEWs, FGD]

## Discussion

### Main finding of the study

Results from this study show that integrating an mHealth solution, with a strong engagement and support strategy, can improve the quality and timeliness of data collection by HEWs for MH and TB in Sidama Zone. The decision to involve HEWs, their supervisors, as well as focal points at health centres, district and zonal levels, allowed the intervention to examine different forms of accountability that can be strengthened through improved data collection, management and use. For example, use of alert messages and related follow up activities by HEWs strengthened accountability to the community; greater communication and support between HEWs and their supervisors strengthened bi-directional accountability; and engaging with decision-makers at different levels of the health system strengthened their ability to use data to improve accountability of planning activities.

The mobile HMIS improved the completeness and quality of data in the intervention areas. HEWs using the system felt a greater sense of opportunity and power to serve their clients better, as well as additional responsibility to manage their time, workload and the smartphones. Findings also pointed to the need for continued attention placed to the ethical dimensions of distributing smartphones, understanding the socio-cultural, gender and financial implications of their use, and reconciling active follow-up of clients with respectful means to understand reasons for non-adherence.

The Ethiopian HEP has been designed to support and strengthen the equity and reach of the health system and is widely heralded as a successful approach.^2^ The HEWs who were using the mobile HMIS were overall very positive about the impact of mHealth on their work, the skills they had developed, their relationships with the community and in their ability to appropriately follow-up with patients.

### What is already known on this topic

The intervention introduced a new approach to data capture, data flow and data sharing at different levels of the health system. This inevitably produces some teething challenges and support and follow-up was required. Existing literature demonstrates that technologies are embedded within existing social, cultural, economic and political structures.^14,15^ MHealth interventions require changes in the behaviours of service providers (HEWs) and their patients (community members).^15^ These changes are driven by social, cultural and environmental factors and as such, they require careful sensitization and customization to have the intended positive impacts.

Research on CHWs’ use of mHealth tends to focus on health outcomes or health system benefits of pilot initiatives.^16–19^ To this end, mHealth has been piloted for use with HEWs and midwives as a data collection tool in Northern Ethiopia.^20^ Reporting on their intervention, Little *et al.* found an improvement in the access of data and that ownership of smartphones is a strong motivator for HEWs.^21^ Echoing a sentiment that was also expressed by some HEWs in our study area, a systematic review by White *et al.*^18^ also found that mobile HMIS that do not allow CHWs to leave blanks in registration forms do not speed up data entry and rather, add to CHWs work burden as they are forced to be more thorough.

### What this study adds

MHealth is arguably an important approach to supporting and empowering HEW to better provide integrated services and feed information and priorities from communities into decision making processes. However, the experiences of the HEWs and the technology influences on their workload and experiences has been less well examined. Our findings highlight that HEWs have the technical capacity to use electronic data capturing mechanisms to improve client follow-up, but that consideration should be given to addressing the ethics and equity of mHealth interventions to better support HEWs. Firstly, efforts are required to ensure mHealth reduces rather than increases workload of HEWs. In this case the mHealth process focused on two priority health areas only rather than the 16 health packages within the remit of HEWs. Hence, they had to simultaneously use mHealth and paper-based reporting. Future steps to expand mHealth across the whole package of work should alleviate this burden. Other challenges in the project related to only one of a pair of HEW situated at the health post receiving a phone. Again, where possible, ensuring all HEWs are appropriately equipped, so they have an opportunity to practice their training and build their skills, would ensure the benefits are appropriately shared. Clear follow up training and shared agreements on what to do in the case of theft or loss or leaving the role of HEW, that do not penalize HEWs is necessary.

### Limitations of this study

This article summarizes results from a mixed method process evaluation. It captured data and perspectives in real time, which limited its ability to adopt a long-term perspective. We do not yet know how, or if, the mHealth process will be sustained through time or what its implications may be on responsive planning and governance. Ensuring ongoing process evaluation using both quantitative data, and the qualitative perspectives of the HEWs (and the health systems and communities they serve) will continue to be important to ensure equity and ethics are meaningfully considered and addressed.

The intervention was also implemented on a relatively small scale, for only two of the 16 essential package areas. As such, while the ongoing paper-based reporting is required, we cannot get a true sense of how the mHealth technology may support HEWs if it was rolled out. Finally, problems related to power failure and connectivity varied by district and not all districts began the intervention at the same time and with equal intensity, which resulted in variations in data collection, reporting and use. As a result, effective comparisons could not be made. Further collaboration with the Ethiopian government, who dominate the telecommunications market in Ethiopia, to improve the network coverage would greatly improve the efficiency of this project and help ensure successful scale-up.

## Conclusion

The mobile HMIS has improved the quality and timeliness of data flow and the responsiveness and impact of the health system. Female HEWs in Ethiopia play a critical interface role between the health system and rural communities. Providing them with smartphones and a responsive mHealth platform, along with focused training and supportive supervision, has enabled this important cadre of health workers to use new technologies and build skills that are positively impacting delivery of health services in their communities. HEWs face large workloads, as is common with many close-to-community providers and we need to do our best to ensure that this new technology supports them in their workload and interactions with community members rather than bring new burdens. An equity and ethics lens is critical for mHealth technology to ensure that new approaches appropriately support staff whose job it is to use these new technologies; and result in better services to the communities they serve.

## Supplementary Material

Supplementary Data

Supplementary Data

## Appendix 1: Further information on process evaluation:

The project staff conducted regular supportive supervision to the districts, health centre, health posts and households to ensure successful implementation of the project and learn from the implementers about the contribution of the project to improve the health system. This provided for further process evaluation through the following channels:

- **Training and sensitization workshops** were conducted with key stakeholders to create awareness of the project and its potential to improve health service delivery. Training was also conducted with HEWs and health staff on HMIS.
- **Regular supportive supervision of HEWs** was conducted by project staff on a weekly basis alongside a review of their activities. The forum was meant to discuss the implementation and use of mobile phones, performance and challenges encountered. In addition, the enrolment of patients and clients to care was monitored in the community and follow up plan was designed to improve the project implementation.
- **Ongoing stakeholder meetings** occurred throughout project period (20× catchment health centre meetings, 2× district meetings, 2× province meetings and 2× national level meetings) to enable experience-sharing, debates and discussions on the use of mobile phones to support health care; further customization of the results and lesson learned and provided fora to discuss future directions on the utilization of mHealth on other health care activities.
- **HEW meetings** were held within districts to ensure HEWs were given opportunity to share their experiences in a comfortable environment. The meetings occurred over twenty times throughout the course of the project with the aim of:
  - Evaluating each district’s mHealth activities.
  - Sharing knowledge and experience of mHealth activities.
  - Provide explanation for, or fill any gaps in data collection.
  - Evaluate the strengths and weaknesses of mHealth activities for each district.
  - Check the performance of district supervisors.
