# Supplementary figures and images for: Can mHealth improve timeliness and quality of health data collected and used by health extension workers in rural Southern Ethiopia?
Source: J Public Health (Oxf). 2018 Dec 14;40(Suppl 2):ii74–86. doi: 10.1093/pubmed/fdy200 (PMC6294041; doi:10.1093/pubmed/fdy200)

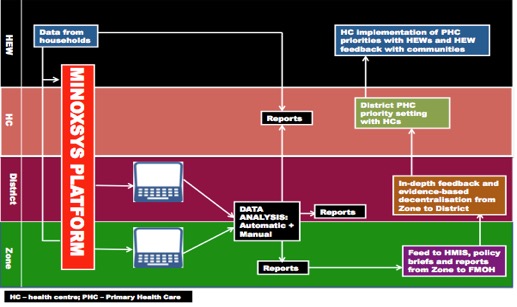

Supplement: Supplementary Data [file fdy200_diagram_2-_platform_of_data_generation.jpeg]

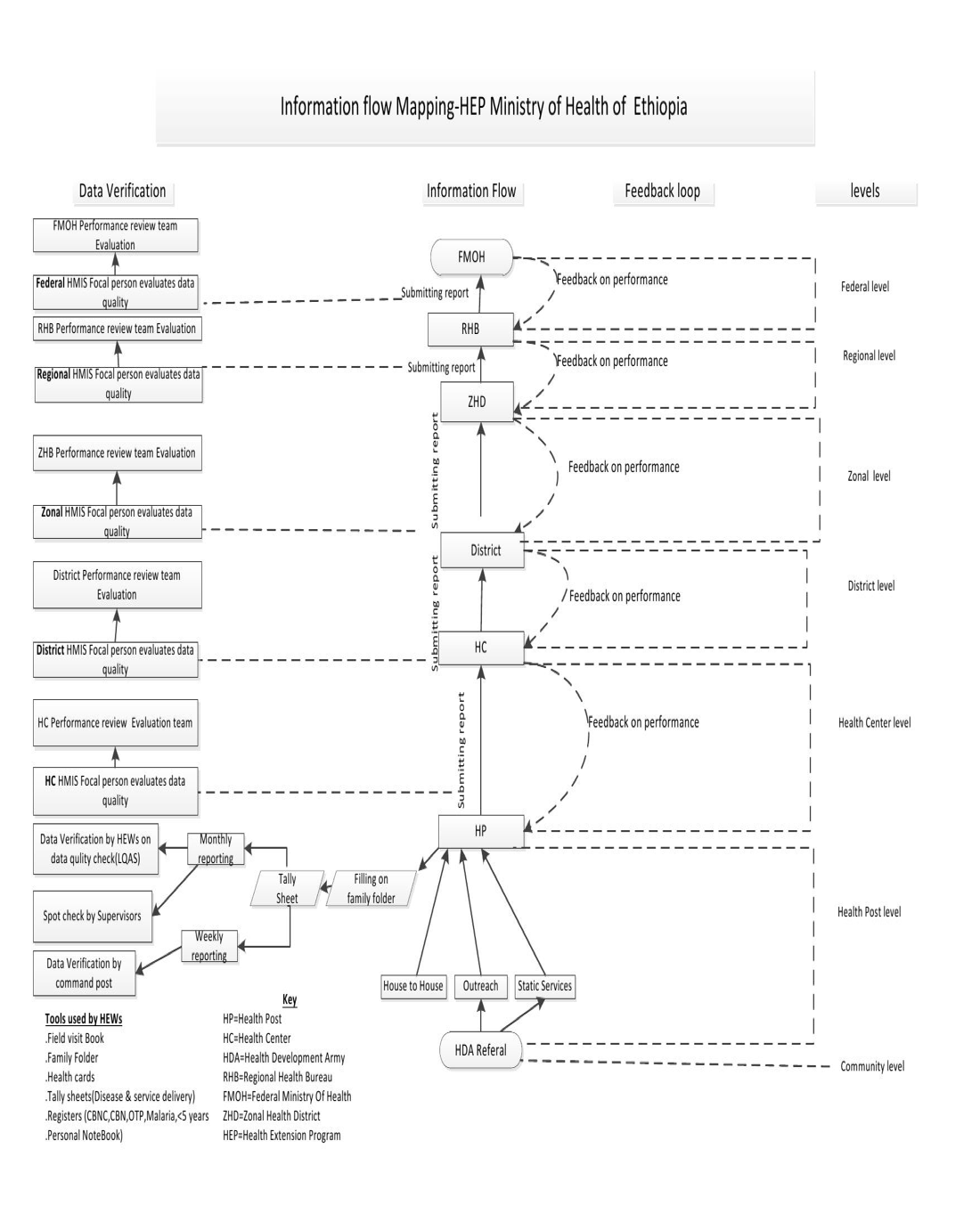

Supplement: Supplementary Data [file fdy200_diagram_3-_information_flow_for_the_health_extension_programme.png]
